# Maternal undernutrition and cardiometabolic disease: a latin american perspective

**DOI:** 10.1186/s12916-015-0293-8

**Published:** 2015-03-02

**Authors:** Patricio Lopez-Jaramillo, Diego Gomez-Arbelaez, Aristides Sotomayor-Rubio, Daniel Mantilla-Garcia, Jose Lopez-Lopez

**Affiliations:** 1 Dirección de Investigaciones, Fundación Oftalmológica de Santander – FOSCAL, Torre Milton Salazar, Primer piso, Calle 155A N. 23-09, El Bosque, Floridablanca, Santander Colombia; 2 Instituto de Investigaciones MASIRA, Universidad de Santander – UDES, Bucaramanga, Colombia; 3 grid.252609.a0000000122968512Escuela de Medicina, Universidad Autónoma de Bucaramanga – UNAB, Bucaramanga, Colombia; 4 Departamento de Endocrinología, Escuela de Medicina, Universidad de Santiago de Compostela - España, Santiago de Compostela, La Coruña, Spain

**Keywords:** Obesity, Cardiovascular diseases, Maternal undernutrition, Low-birth weight, Socio-economic inequalities, Latin America, Developing countries

## Abstract

The current epidemic of obesity and cardiometabolic diseases in developing countries is described as being driven by socioeconomic inequalities. These populations have a greater vulnerability to cardiometabolic diseases due to the discrepancy between the maternal undernutrition and its consequence, low-birth weight progeny, and the subsequent modern lifestyles which are associated with socioeconomic and environmental changes that modify dietary habits, discourage physical activity and encourage sedentary behaviors. Maternal undernutrition can generate epigenetic modifications, with potential long-term consequences. Throughout life, people are faced with the challenge of adapting to changes in their environment, such as excessive intake of high energy density foods and sedentary behavior. However, a mismatch between conditions experienced during fetal programming and current environmental conditions will make adaptation difficult for them, and will increase their susceptibility to obesity and cardiovascular diseases. It is important to conduct research in the Latin American context, in order to define the best strategies to prevent the epidemic of cardiometabolic diseases in the region.

## Introduction

Overweight and obesity are defined as a body mass index (BMI) of 25–29.9 and greater than or equal to 30 kg/m^2^, respectively. Worryingly, prevalence is increasing at an alarming rate throughout the world and it has been projected that in 2030, there will be 2.16 billion overweight and 1.12 billion obese people in the world [1]. Developing countries have seen a proportionally larger increase in the number of overweight and obese individuals. For instance, some years ago it was reported that a third of the population in Latin America were overweight or obese [2,3], whereas current data suggests that it has increased to around half of the adults [4].

This trend is thought to be driven by the rapid and unequal socio-economic development that Latin America is experiencing. Significant nutritional changes, including increases in high energy density foods consumption, with a parallel decreases in physical activity levels, due to the mechanization of both daily work and leisure-time activities [5-8], are being observed. Moreover, the migration from rural to urban areas could be also contributing to these lifestyle changes [9].

Despite the increased prevalence of obesity and overweight, maternal undernutrition and its consequence, low-birth weight progeny, remains an important public health concern in many developing regions, such as Latin America [10]. This condition may be also a consequence of the unequal socio-economic development observed in the region, since both maternal undernutrition and placental dysfunction induced by preeclampsia or infections, can lead to intrauterine growth restriction (IUGR) [11]. Indeed, socio-economic inequality is a determinant of poor access of pregnant women belonging to a low socio-economical stratum to an adequate diet and to an appropriate prenatal care [10,12], in turn risk factors for the mentioned placental disorders, and hence, for IUGR.

Interestingly, it has been demonstrated that a poor nutrition during the fetal development and early in the extra uterine life is associated with increased risk of cardiometabolic disease in adulthood [13,14]. Therefore, the main goal of this review is to explain, from our point of view, how the socio-economic inequality mediated by a mismatch between maternal undernutrition and exposure to westernized lifestyles in later life is contributing to the current increasing prevalence in obesity and cardiometabolic diseases in the Latin American population. Moreover, we will review the association between these trends and epigenetic adaptations, insulin resistance and low-degree inflammation.

## The Cardiometabolic disease situation in Latin America

There have been large shifts in the morbi-mortality trends in Latin America in recent decades, moving from a dominance of transmissible to a dominance of non-transmissible chronic diseases, including cardiovascular and metabolic diseases [2,5,9,15]. For example, while national, regional and global trends in fasting plasma glucose and diabetes prevalence since 1980 [16], show that glycemia levels and type 2 diabetes mellitus (DM2) are an increasing hazard worldwide (Table 1); higher prevalences are reported in some regions, including Oceania and Latin America. In fact, the estimated increase in the number of people with DM2 in Latin America will be over 150% in just three decades, from 15 million in 1995 to 39 million in 2025 [17]. Results of small studies confirm these exceptionally high prevalences of DM2, such as values as high as 43.3% in Puerto Rico [18]. The lowest prevalence has been reported in Peru (5% in Lima) [19].

**Table 1:** **Global trends in fasting plasma glucose and diabetes prevalence (1980 vs. 2008)**

**Variables**	**1980**	**2008**
Global FPG (mmol/L)*		
Men	5.29**	5.50 (5.37–5.63)
Women	5.15**	5.42 (5.29–5.54)
DM2 prevalence (%)*		
Men	8.3 (6.5–10.4)	9.8 (8.6–11.2)
Women	7.5 (5.8–9.6)	9.2 (8.0–10.5)
People with DM2 (million)*		
Men	77 (60–97)	173 (151–197)
Women	76 (58–97)	173 (151–197)

*Age-standardized values. **Estimated values from the published data [16]. Data is presented as mean (95% uncertainty interval). FPG: fasting plasma glucose. DM2: diabetes mellitus type 2.

In relation to obesity, a systematic analysis reported by the Global Burden of Metabolic Risk Factors of Chronic Diseases Collaborating Group demonstrated that between 1980 and 2008, the mean BMI increased worldwide by 0.4 kg/m^2^ per decade for men and 0.5 kg/m^2^ per decade for women. In Latin America the largest rise in BMI occurred in females, in which rises of 1.3 kg/m^2^ per decade were observed [20]. In global terms it is clear that the poorest countries in Latin America, such as Haiti, Honduras and Bolivia, have the lowest rates of obesity. However, similar rates of increase are observed in these countries as in the region as a whole [1,3], being higher in women, and disproportionately affecting groups from lower socio-economic strata who are living in urban areas [3].

Moreover, the metabolic syndrome (MetS) has been always considered as a major public health concern. It was first described predominantly in developed countries such as the United States, where a prevalence of 24% in the adult population was reported [21]. Nonetheless, various further studies in Latin American countries [19,22-28], have demonstrated that currently these countries have a similar or even higher prevalence of MetS in adults than developed countries (Table 2).

**Table 2:** **Prevalence of metabolic syndrome in Latin American countries**

**Country**	**Year**	**Participants (n)**	**Age (years)**	**Diagnostic criteria**	**MS prevalence (%)**
Chile	2003	1833	≥17	ATP-III	32.0
				IDF	37.0
Mexico	2006	6021	20 to 69	ATP-III	36.8
				IDF	49.8
Venezuela	2001	3108	≥20	ATP-III	31.2
Ecuador	2004	352	≥65	IDF	40.0
Peru	2006	1878	20 to 80	AHA/NHLBI	18.8
Brazil	2001	1655	25 to 64	ATP-III	32.9
Colombia	2007	1001	≥18	ATP-III	45.6
				IDF	50.4

Extracted values from published data [19,22-28]. MS: metabolic syndrome.

Less data is available about the prevalence of MetS and its components in children and adolescents in Latin America. A small study of children and adolescents of Bolivia reported a high frequency of MetS (36% of the sample) [29], and in another recent study, in Chilean adolescents 37.5% presented with MetS [30]. Therefore, while malnourishment remains a large problem in a substantial proportion of poor Latin American children, obesity is emerging in alarming rates, principally in the most developed sectors in these countries [8]. For example, we recently reported a lower prevalence of undernutrition (4.4%) in school age Colombian children than of overweight (12.9%) and obesity (9.8%) [31].

The non-communicable chronic disease situation in Latin America is one that is of concern not only in terms of prevalence, but also due to its low rates of awareness, treatment and control. The Prospective Urban and Rural Epidemiology (PURE) study, in which 4 Latin American countries (Argentina, Brazil, Colombia and Chile) are currently participating, recently reported worrisome global and regional rates of awareness, treatment and control of hypertension [32], as well as a very low use of the proven effective secondary preventive drugs in those patients with a previous history of coronary heart disease or stroke [33]. Moreover, among these individuals with self-reported coronary heart disease or stroke, the prevalence of a healthy lifestyle was also very low (Table 3) [34]. The Latin American situation is of concern when compared to high-income countries. The above situations are other examples of the poor access to adequate health care in our population, so as it is prenatal care for mothers.

**Table 3:** **Global and Latin American rates of awareness, treatment and control of cardiovascular diseases – PURE study**

**Variables**	**Global prevalence (%)**	**Latin American prevalence (%)**
Hypertension		
Awareness	46.5	57.1
Treatment	40.6	52.8
Control	13.2	18.8
Secondary preventive drugs		
Antiplatelet drugs	25.3	29.0
β-blockers	17.4	28.8
ACE / ARB	19.5	37.9
Statins	14.6	15.0
Healthy lifestyle		
Smoking cessation	53.4	67.2
Physical activity	35.1	41.5*
Healthy diets	39.0	43.2*

Extracted values from published data [32-34]. *Data for the global lower-middle-income countries, where the Latin Americans are included. ACE: angiotensin-converting-enzyme inhibitors. ARB: angiotensin-receptor blockers.

In this context, the social and financial costs of obesity and cardiometabolic diseases are also increasing at excessively high rates. Certainly, premature mortality and temporary and permanent disability generated as complications of non-communicable chronic diseases represent an enormous burden for patients and their families, as well as for the health system and society in general [35]. For example, obesity has been shown to account for up to 16% of the global burden of disease, expressed as a percentage of disability-adjusted life-years. Moreover, there has been described that around of 10% of total health care costs in the developed world are attributable to obesity [36]. This is especially worrisome in emerging economies, such as Latin Americans.

Several strategies have been implemented to reduce the burden of cardiovascular diseases (CVD) in Latin America. For instance, recently the Latin American consensus on hypertension in patients with DM2 and MetS was published [37] to serve as a guide for physicians taking care of patients with these diseases and comorbidities. In addition, the Latin American Society of Hypertension (LASH) defined ‘the initiative 20/20’ as an institutional goal, which aims to improve the awareness, treatment and control of hypertension in 20% by the year 2020 [38]. Unfortunately, all these important actions are only mitigating the problem, but do not address the causes of non-communicable chronic diseases. We therefore believe that several public health policies should be adopted which are aimed at reducing social inequality in our region, since it is underlies the link between early life conditions, including intrauterine, and the regional epidemic of cardiometabolic diseases in later life, as we are going to review below.

## Maternal undernutrition, epigenetics and cardiometabolic diseases

Inter-population differences in the prevalence of cardiovascular and metabolic diseases and its associated risk factors may reflect differences in the quality of life status between them, which could be influenced by several environmental factors, such as physical activity and dietary patterns. However, genetic background might also play a key role, since the genome is programmed to express appropriate sets of genes, in particular tissues, at specific time points during the individual’s life. Even more important however, may be the genetic-environment interaction in the pathogenesis of cardiometabolic diseases, which can induce epigenetic modifications [10,13,14].

By definition, epigenetics refers to modifications in gene expressions that are controlled by changes in DNA methylation and/or chromatin structure [39], and might play an important role in the pathogenesis of various entities, including cancer and cardiometabolic diseases. Epigenetic events are heritable, although occasionally reversible, depending on endogenous, but specially exogenous (environmental) signals, creating a memory of cell identity [40], and maintaining genomic functions after differentiation, propagation of essential features of chromosomal architecture, and dosage compensation [41]. Therefore, epigenetic modifications could lead to irreversible processes of differentiation and organogenesis or to labile and potentially reversible changes in homeostatic processes [14]. Moreover, environmental signals such as food depletion and stress have been present throughout evolution, and organisms have had to sense and adapt to them, to ensure their survival. These epigenetic modifications occur not only in humans, but have also been implicated in the control of various parameters of gene expression, genetic recombination, DNA repair, and DNA mutagenesis in bacteria, plants, and animals [42].

Epigenetic mechanisms include DNA methylations, histone modifications, and microRNAs [43], and can help to explain how individuals with identical or similar DNA, when exposed to different environmental signals, express diverse phenotypes and differ in their susceptibility to certain pathologies. Thus, chromatin structure can be linked to environmental factors such as diet, nutrients, drugs or the socio-economic environment in several ways [40,44]. From this perspective, we propose that the very rapid change from undernutrition to overnutrition in Latin American and the Caribbean countries [3-5], a consequence of the socio-economic transition process, may be producing an epigenetic maladaptation in these populations.

**Figure 1:**
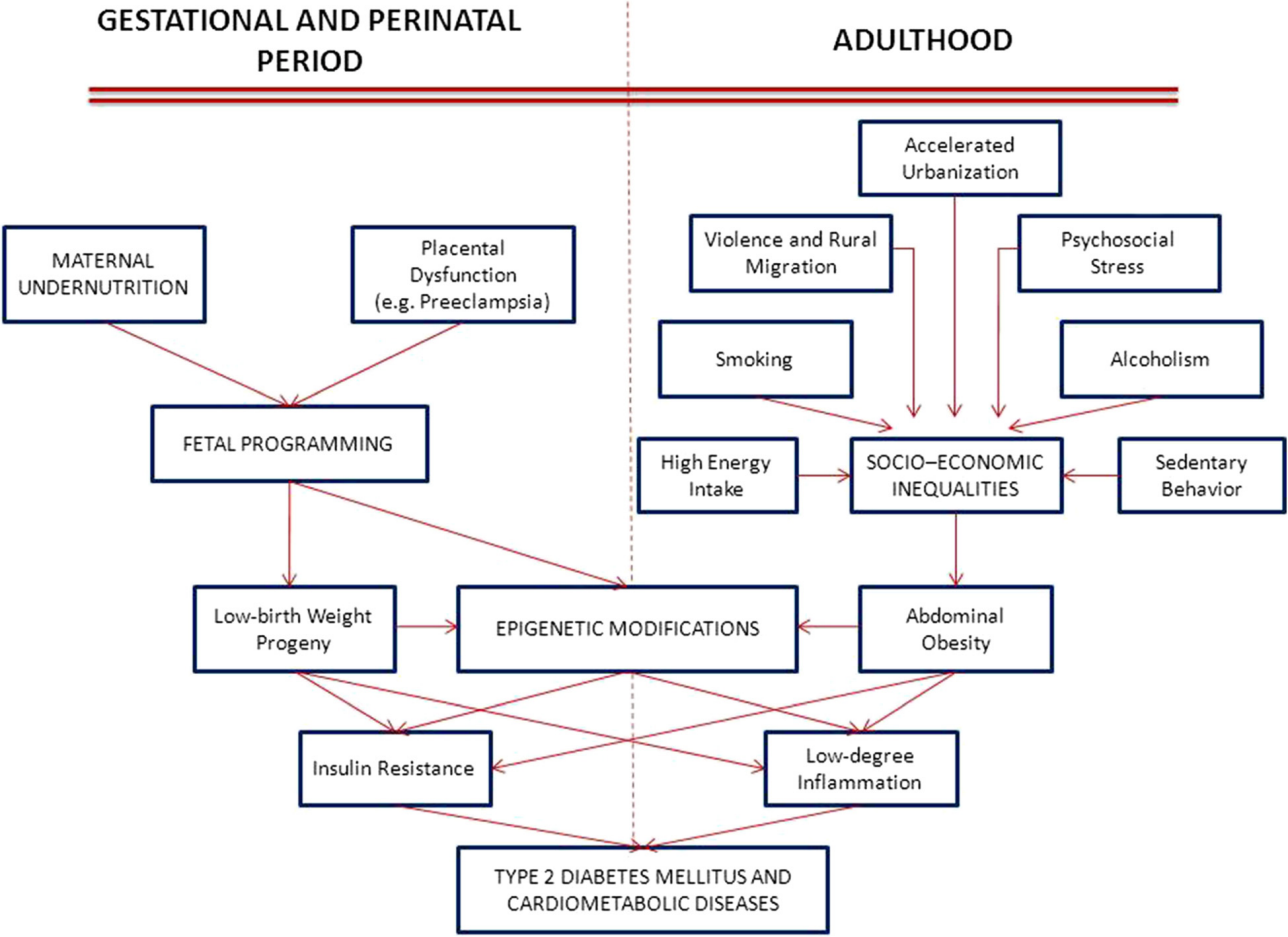
**Epigenetic modifications and environmental influences on the pathogenesis of cardiometabolic diseases.**

There is an interesting discrepancy between developed and developing countries, and the causal direction of the relationship between socio-economic conditions and obesity and CVD is complex. In developing countries, at the start of the epidemiologic and nutrition transition, a higher socio-economic level was associated with a more unhealthy diet and higher levels of obesity, while over time a gradual shift and reversal of this relationship has been observed. Currently, higher socio-economic status is associated with knowledge of good nutrition and lifestyle choices and an inverse relation between socio-economic levels and obesity is being observed, as found in developed countries. Meanwhile, later in the transition process, poorer people in developing countries have adopted increasingly unhealthy lifestyles and changes in their nutritional habits [5,7,8], trends currently occurring in the Latin American population. Thus, in these populations, westernized habits of high energy intake and low energy expenditure during adulthood contrast substantially with the epigenetic fetal programming based on maternal conditions of undernutrition, thereby mediating an increased susceptibility to high incidence of cardiovascular and metabolic diseases (Figure 1).

During early human and animal development, exogenous or environmental signals and changes could induce epigenetic modifications, which have potential long-term consequences [40,45]. In some populations, poor maternal nutrition has been associated with increased risk of DM2 over several generations [14,46]. Moreover, placental insufficiency, inadequate maternal nutrition, metabolic disturbances or neonatal medication can induce incorrect epigenetic programming during early development, which could partially explain lifelong imbalance between energy intake and energy expenditure in individuals with MetS, obesity, DM2 and CVD [14,46-51].

Indeed, human epidemiological studies and appropriately designed dietary interventions in animal models have provided considerable evidence to suggest that maternal nutritional imbalance and metabolic disturbances, during critical time windows of development, may have a persistent effect on the health of offspring and may even be transmitted to the next generation [14,47,52-57]. As a consequence, the hypotheses of “fetal programming” and its new denomination: “developmental origins of health and disease” were proposed, describing the early nutrition during gestation and lactation as a common risk factor for chronic diseases, such as obesity, CVD, diabetes, hypertension, asthma, cancer and even schizophrenia [58-66].

Furthermore, cohort studies after famine also suggest that several adulthood diseases are related to the duration and timing of nutritional deficit during the gestational period. As epigenetic plasticity changes continuously from conception to death, effects will vary according to whether the exposure occurs during preconception, pregnancy, lactation, neonatal life, early life, pre-/post-menopause, or puberty [53]. Furthermore, several other processes, such as chromosomal instability, telomere shortening, metabolic cycles, mitochondrial deteriorations, and oscillatory, circadian or seasonal rhythms of systemic hormone levels (hypothalamic–pituitary–adrenal axis), could also affect epigenetic plasticity [50,52,58,59,67-70]. In principle, the earlier the epigenetic changes occur, the greater their physiologic/metabolic impact such that epigenetic modifications that occur during embryogenesis and early fetal development would be transmitted over consecutive mitotic divisions, affecting many more cells than those occurring during postnatal development [55]. Data from the Dutch Winter Hunger cohort revealed that those descendants of mothers who were severely undernourished during the early stage of pregnancy were more likely to develop CVD than those born to mothers whose pregnancies were more advanced at the time of nutritional deficit [71-73].

Various conditions and nutritional restrictions during pregnancy have been linked with a number of different metabolic outcomes on their offspring in humans and animals. Low maternal protein consumption or poor vitamin B and methionine status are associated with behavioral and cardiovascular abnormalities, and sex-specific changes in hepatic gene expression in rat fetuses and changes in imprinted gene expression in the rat embryo-fetal axis [40,74-76]. Katari et al. [77] highlighted the association between in-vitro conception and changes in DNA methylation, in turn affecting the long-term pattern of expression of genes involved in chronic metabolic disorders such as obesity and DM2. Pinney and Simmons [78] studied epigenetic events at the promoter of the gene encoding Pdx-1, a critical transcription factor (TF) for beta-cell function and development, the expression of which is reduced in intra uterine growth restriction (IUGR), a situation that has been associated with the development of diabetes in adulthood [15,40,78]. As mentioned above, IUGR can be a consequence of maternal undernutrition and can predispose to the development of DM2 in the new born when exposed to different, energy-rich diet in later life [10,13]. Raychaudhuri et al. [79] focused on the sequence of epigenetic mechanisms responsible for the weak expression of Glut-4 in the skeletal muscle of individuals with IUGR. They found that perinatal nutrient restriction resulting in IUGR leads to histone modifications in skeletal muscle that directly decrease Glut-4 gene expression. This effectively creates a metabolic knockdown of an important regulator of peripheral glucose transport and insulin resistance, thereby contributing to the adult DM2 phenotype [79].

In addition, it has been demonstrated that IUGR may be related to such vascular abnormalities as stiffness of the abdominal aorta [80], reduced arterial compliance [81], narrower retinal arteriolar caliber [82], and endothelial dysfunction [83], factors which also contribute to the development of CVD later in life. Maternal protein restriction during pregnancy may also result in a declined skeletal muscle mass of the progeny [84]. In a recent study it was shown in IUGR pigs that their total number of muscle fibers was lower when compared with normal body weight animals [85]. Furthermore, there was altered the expression of 37 proteins involved in the proliferation and differentiation of muscle fibers, energy supply, protein metabolism, nutrient transport, intracellular environment, and tissue integrity. We recently demonstrated in a population of Colombian children from low socio economical strata, an association between low muscle strength and increased levels of adipocytes, C-reactive protein (CRP), HOMA index and metabolic risk factors [31]. Moreover, in a sub-analysis of the ORIGIN study [86] we demonstrated that low handgrip strength is an important factor associated to an increased risk of cardiovascular mortality in prediabetic and diabetic patients.

Furthermore, even pre-implantation development in mammals has recently been shown to be sensitive to environmental conditions [87]. Results of both in-vivo and in-vitro experiments, have demonstrated that environmental factors can modify blastocyst potential and lead to long-term changes in fetal and postnatal health and physiology. For example, elevated concentrations of plasma homocysteine have been found in pre-implantation embryos, which have low mtDNA copy number and subsequently develop DM2 [88]. Similarly, the environment inhabited by breeding females before conception and early in pregnancy has striking effects on the oocytes developing in the ovarian follicle and embryos in the early stages of development in the reproductive tract. Environmental conditions at these stages may also alter behavior, cardiovascular function and reproductive function throughout postnatal life [40,89-92].

Consequently, fetal and neonatal periods are critical for the development and growth of the systems involved in cardiometabolic pathways. In rats, detrimental effects on growth during fetal and early postnatal life can negatively affect both the number [93] and secretor function of pancreatic beta-cells [94]. Some years ago, Hales and colleagues demonstrated that men with a low-birth weight were six times more likely to have DM2 at 64 years of age than men with a high-birth weight [95]. Moreover, it has been demonstrated that children who had a low-birth weight but had increased rates of growth at 7 years old had a further increased risk of developing DM2 later in life [96]. Hence, it is interesting to propose that the increased rates of cardiovascular and metabolic diseases, currently observed in Latin America could be the result of the discrepancy between the restricted nutritional environment during fetal development and early life, and the environment of nutritional abundance during adulthood. This discrepancy causes a mismatch between the fetal programming of the organism’s metabolic pathways and their adult circumstances, characterized by the imposition of new obesogenic lifestyles [5,10,13,15].

Moreover, recent studies have found significant differences in DNA methylation profiles in aged monozygotic twins with a history of non-shared environments, suggesting that environmentally mediated epigenetic changes also occur throughout life [97,98]. For example, important CVD risk factors such as hypercholesterolemia, obesity, hyperhomocysteinemia, and hyperglycemia could stimulate the inflammatory process and its long-term effects via epigenetic reprogramming, promoting differentiation of monocytes/macrophages into more pro-atherogenic phenotypes [99-102].

## Abdominal obesity, insulin resistance and low degree inflammation

Overweight and obesity have been well described as important risk factors for CVD [12,103,104] and DM2 [3,5,103], and to be associated with an increased prevalence of other cardiovascular risk factors [103]. Recently, in Colombian adults with severe coronary disease, we reported that abdominal obesity is associated with leptin/adiponectin imbalance, decreased endothelium-dependent relaxation and an enhanced response to angiotensin-II. These changes occurred independently of other cardiovascular risk factors and therefore suggest that these vascular alterations, promoted by abdominal obesity, could be the initial event that leads to insulin resistance, low degree inflammation, atherosclerosis and CVD [104].

Moreover, the INTERHEART [105] and the INTERSTROKE [106] studies, which included Latin American countries (Argentina, Brazil, Chile, Colombia, Ecuador, Peru), demonstrated that abdominal obesity, evaluated by waist-to-hip ratio, was a more sensitive risk factor than BMI in subjects that presented a first event of myocardial infarction and stroke. Furthermore, abdominal obesity is considered as the key factor in the onset of MetS, a cluster of hypertension, dysglycemia, low HDL cholesterol, increased triglycerides, and abdominal obesity. MetS is also related to an increased risk of DM2 and CVD [107], an important consideration given that abdominal obesity is highly prevalent in Latin America.

A recent report of the International Day for Evaluation of Abdominal Obesity (IDEA) study [108], showed that the mean waist circumference in a Latin American primary care population was 96.4 cm and 89.7 cm for men and women, respectively. This study also confirmed the association between increased abdominal obesity and the presence of DM2 and CVD in our population. However, the cutoff point to diagnose abdominal obesity in the Latin American population remains controversial. A study conducted in Colombian subjects with no previous CVD history, reported that the criteria for waist circumference proposed by the International Diabetes Federation (90 cm: men, 80 cm: women) is more useful for identifying subjects with MetS than that proposed by the Adult Treatment Panel III (102 cm: men, 88 cm: women) [22]. In addition, several studies [109,110] carried out in developing countries have reported lower waist circumference cutoff points for cardiovascular risk than those reported in developed countries. In healthy young Colombian men, a waist circumference of 88 cm identified subjects with cardiovascular risk with a sensitivity of 83.7% and a specificity of 84.8% [111]. In Ecuador [112], it was demonstrated that a waist circumference of 90 cm in men is the best cutoff point associated with the presence of at least two of the other MetS criteria according to the Adult Treatment Panel III.

Recently, it has become evident that the visceral adiposity content is crucial in determining risk of developing DM2 and CVD [113]. Waist circumference was reported to be an easy to implement measure for evaluating the content of visceral fat, which is the main source of pro-inflammatory cytokines [113-116]. Moreover, some studies have observed that the concentration of pro-inflammatory cytokines is higher in the Latin American population than that reported in the population of developed countries, suggesting a higher sensitivity of this population to develop systemic low-degree inflammation in response to abdominal obesity [117,118]. These cytokines are elevated in the serum of obese subjects [119] and it has been proposed that the systemic inflammation produced by the adipose tissue participates in all stages of the development of cardiometabolic diseases, such as endothelial dysfunction [120], atheroma formation, rupture of plaque, and acute thrombotic complications [121,122]. C-reactive protein (CRP), produced by the liver in response to the stimulus of TNF-alpha and interleukin-6, is increased in subjects with multiple acute coronary events and is a strong independent predictor of new acute coronary events [123]. We have demonstrated in the Andean region that CRP is an independent risk factor for essential hypertension [124] and preeclampsia [125,126]. Moreover, the concentration of CRP is increased in dyslipidemic subjects with MetS [127] and in overweight children [128].

All these studies confirm that Latin Americans have an increased risk of developing cardiometabolic diseases at lower levels of abdominal obesity. We propose that this observation could be mediated by epigenetic modifications acquired during fetal development, which could affect visceral adipose tissue and predispose to an inflammatory imbalance. Recently, we found evidence of regional differences in adiponectin levels in subjects with MetS, especially between developed vs. developing countries [129]. However, we were not able to ascertain whether the lower adiponectin values observed in subjects with MetS from developing countries were related to genetic and or epigenetic factors. Therefore, additional studies are required to further test this hypothesis.

Inflammation has been described as an adaptation to the disruption of homeostasis at a cellular and tissue level and affects many important processes, such as host defense, tissue remodeling and repair, and the regulation of metabolism. All of processes related to the inflammatory response require coordinated control in some conditions, and independently in others [130,131]. This is accomplished with the participation of several mechanisms that operate at different levels, including alterations in the composition of immune cells in tissues, changes in cell responsiveness to inflammatory stimuli, regulation of signaling pathways and epigenetic control of gene expression [66].

For example, signal-specific mechanisms operating at the molecular level can activate the key transcription factor (TF) nuclear factor-kB (NFkBTF), which is probably the most important TF and mediator of inflammation, since it controls the expression of more than 400 genes [132-134]. In addition, NFkBTF is involved in increasing inflammatory disease and malignancy by inducing transcription of soluble mediators that amplify inflammation, angiogenesis and neoplastic cell proliferation, and promoting progression to more aggressive disease states [135]. Moreover, infectious agents and over nutrition, specifically via metabolic and endoplasmic reticulum (ER) stress, are some of the numerous stimuli that can activate to NFkBTF family. Indeed, a considerable constitutive activity of NFkBTF and its associated processes has been observed in many cancer cells, inflammatory disorders, obesity and insulin resistance [132,133,136-143].

Obesity is characterized by a chronic exposure to high energy intake and positive energy balance, and ER is the organelle responsible for responding to these challenges. ER acts as a key nutrition sensor of cellular metabolic parameters, such as hyperglycemia, fatty acid overload, hypoglycemia, and oxidative stress, and participates in almost all anabolic and catabolic processes. Therefore, an eventual failure of the ER’s adaptive capacity would affect many different inflammatory and stress signaling pathways at the crossroad of inflammation, cancer and metabolic disease [138,139,144].

Besides the molecular and cellular levels, there are some gene-specific mechanisms operating at the level of individual genes and gene subsets. Several TF and extragenic noncoding RNAs participate in the induction of inflammatory transcriptional responses by acting on inflammatory enhancers [145-148]. Hence, a well-ordered expression of cytokine genes is a crucial component of an immune response and is decisive for homeostasis. Various factors, including the type of cytokine, as well as the cell type, dose range and the kinetics of its expression are extremely important to generate an appropriate response to metabolic stress or an infectious condition [149-151]. Recently, several efforts have been made to modulate epigenetic factors, such as via hyper/hypomethylation of key inflammatory genes by external and dietary factors, aiming to cure or protect against inflammatory disease [53,152-156].

Different cell types constitutively express primary-response TF in their cytoplasm and these are activated by signal-dependent post-translational modifications, which involve their nuclear translocation, such as NFkB, IRF, and CREB. These transcription factors are mainly responsible for the primary phase of gene induction and integrate signals from diverse signaling pathways that can amplify or terminate signal-dependent TF activation. Other classes of TF, such as C/EBPd, require *de novo* synthesis following inflammatory stimulation. Most are constitutively nuclear and regulate secondary waves of gene expression. Other class of constitutively nuclear TF is expressed in a cell type-specific and differentiation-dependent manner, such as Runx, PU.1, IRF8, AP1, and C/EBP [146,157]. They establish cell type-specific patterns of gene expression and are involved in chromatin remodeling during cell differentiation and organization of high-order chromatin structure and chromosomal domains. The TF of these categories do not act independently, but function coordinately to control the inflammatory transcriptional response. Upon combining datasets of expression profiling of inflammatory genes and in silico motif scanning of promoters of these genes they can define gene clusters that are coordinately regulated and the TF that are likely to control their expression [130,132,134,141,149,157-160]. As TF bind very poorly, or not at all, to nucleosomal DNA, their activation is therefore coordinated to the recruitment of ATP-dependent chromatin-remodeling factors, histone-enzyme complexes, methylases, demethylases, acetylases, and deacetylases, amongst other substances. Parallel post-translational modifications such as phosphorylation, acetylation, methylation, ribosylation, sumoylation, and ubiquitination of histone and non-histone TF and cofactor complexes, permit formation of dynamic enhanceosome complexes which establish a distinct chromatin structure. Ultimately, all of these epigenetic reactions and modifications are the decisive step in which both environmental and differentiative inputs determine the correct or incorrect expression of each inflammatory gene [66,132,158,160-162].

## Conclusion

Socioeconomic inequalities are emerging as an important determinant of the current worldwide epidemic of obesity and CVD. While the relationship between socioeconomic conditions and obesity and CVD is inconsistent in developed countries, in developing countries there appears to be a strong association between the two. As a possible explanation, it is suggested that in developing countries maternal disadvantage leads to a low-birth weight as a consequence of poor health behaviors, exposure to harmful environmental factors, poorer access to medical care, and less worse underlying maternal health. Later in life, due to the socioeconomic transition, these children have been exposed to a greater access to energy-dense diets, and an increased risk of developing obesity and CVD. We therefore argue that a key solution to address this public health concern is the implementation of public policies which aim to reduce socioeconomic inequalities. Despite having achieved some important goals, it is noteworthy that we are fighting a huge enemy - the increasing epidemic of CVD driven by our social inequalities. Therefore, the great challenge is to continue to develop and implement research in our population which aims to find regional-specific solutions to resolve a worldwide problem.

## Abbreviations

BMI: Body mass index
CRP: C-Reactive protein
CVD: Cardiovascular diseases
DM2: Diabetes mellitus type 2
ER: Endoplasmic reticulum
IUGR: Intrauterine growth restriction
MetS: Metabolic syndrome
TF: Transcription factor
